# Inhibition of human diffuse large B-cell lymphoma growth by JC polyomavirus-like particles delivering a suicide gene

**DOI:** 10.1186/s12967-015-0389-0

**Published:** 2015-01-27

**Authors:** Chun-Nun Chao, Yih-Leh Huang, Mien-Chun Lin, Chiung-Yao Fang, Cheng-Huang Shen, Pei-Lain Chen, Meilin Wang, Deching Chang, Chih-En Tseng

**Affiliations:** Institute of Molecular Biology, National Chung Cheng University, Chiayi, Taiwan; Department of Pediatrics, Chiayi Christian Hospital, Chiayi, Taiwan; Department of Medical Research, Buddhist Dalin Tzu Chi General Hospital, Chiayi, Taiwan; Department of Urology, Chiayi Christian Hospital, Chiayi, Taiwan; Department of Medical Research, Chiayi Christian Hospital, Chiayi, Taiwan; Department of Medical Laboratory Science and Biotechnology, Central Taiwan University of Science and Technology, Taichung, Taiwan; Department of Microbiology and Immunology, Chung Shan Medical University, Taichung, Taiwan; Department of Anatomic Pathology, Buddhist Dalin Tzu Chi General Hospital, Chiayi, Taiwan; School of Medicine, Tzu Chi University, Hualien, Taiwan

**Keywords:** Diffuse large B-cell lymphoma, Gene therapy, JCPyV VLPs, Suicide gene, HSV-TK/GCV

## Abstract

**Background:**

Diffuse large B-cell lymphoma (DLBCL) is one of the most common types of aggressive B-cell non-Hodgkin lymphoma. About one-third of patients are either refractory to the treatment or experience relapse afterwards, pointing to the necessity of developing other effective therapies for DLBCL. Human B-lymphocytes are susceptible to JC polyomavirus (JCPyV) infection, and JCPyV virus-like particles (VLPs) can effectively deliver exogenous genes to susceptible cells for expression, suggesting the feasibility of using JCPyV VLPs as gene therapy vectors for DLBCL.

**Methods:**

The JCPyV VLPs packaged with a GFP reporter gene were used to infect human DLBCL cells for gene delivery assay. Furthermore, we packaged JCPyV VLPs with a suicide gene encoding thymidine kinase (TK) to inhibit the growth of DLBCL *in vitro* and *in vivo*.

**Results:**

Here, we show that JCPyV VLPs effectively entered human germinal center B-cell-like (GCB-like) DLBCL and activated B-cell-like (ABC-like) DLBCL and expressed the packaged reporter gene *in vitro*. As measured by the MTT assay, treatment with tk-VLPs in combination with gancyclovir (GCV) reduced the viability of DLBCL cells by 60%. In the xenograft mouse model, injection of tk-VLPs through the tail vein in combination with GCV administration resulted in a potent 80% inhibition of DLBCL tumor nodule growth.

**Conclusions:**

Our results demonstrate the effectiveness of JCPyV VLPs as gene therapy vectors for human DLBCL and provide a potential new strategy for the treatment of DLBCL.

## Background

Diffuse large B-cell lymphoma (DLBCL) is one of the most common types of aggressive B-cell non-Hodgkin lymphoma (B-NHL) and accounts for about 31% of adult B-NHLs in western countries [1]. Based on different gene expression profiles, DLBCL includes germinal center B-cell-like (GCB-like) and activated B-cell-like (ABC-like) DLBCL [2]. Overall survival of patients with GCB-like DLBCL is better than that of patients with ABC-like DLBCL [3]. Conventionally, the mainstay of multi-agent chemotherapy for DLBCL is the CHOP regimen, consisting of cyclophosphamide, doxorubicin, vincristine, and prednisone [4]. In recent years, the addition of the anti-CD20 monoclonal antibody rituximab to CHOP has led to an improvement in patient survival [5]. However, around one-third of patients with advanced-stage DLBCL will be still refractory to therapy or will relapse after early response to treatment [6]. As the majority of DLBCL patients with relapsed disease or resistance to modern multi-agent therapy will eventually succumb to lymphoma, it is essential to develop novel modalities for DLBCL treatment.

In its basic concept, gene therapy is transferring a piece of genetic material into a target cell for the purpose of curing or slowing the progression of disease [7]. Much progress has been made to date in gene therapy for cancer, which has resulted in many clinical trials [8,9]. Therefore, gene therapy may offer new treatment options for patients with hematological malignancies. Many of the gene therapy approaches have been designed on the basis of known genetic lesions of hematological cancers. A *BCL*-2 gene translocation is found in almost all follicular lymphomas and in some diffuse large cell lymphomas [10,11]. In Burkitt’s lymphoma, a translocation of the *C-MYC* oncogene leads to its overexpression [12]. Accordingly, antisense oligonucleotides specific for *BCL*-2 and *C-MYC* are among the current gene therapy approaches for lymphomas [13,14]. Other gene therapy approaches include those that enhance the immune response through IL-2, IL-12 [15,16], that express the suicide gene herpes simplex virus (HSV) thymidine kinase [17], and that use oncolytic viruses [18]. Although these gene therapy strategies have been shown to inhibit lymphoma growth, the gene transfer efficiency is generally low; thus, the development of more effective gene-transducing strategies is vital.

Virus-like particles (VLPs) are made of viral proteins and structurally resemble viral capsids, but do not contain viral genetic materials; yet VLPs have similar infectious pathways as virions [19,20]. The ability of VLPs to package nucleic acids makes them promising vectors for gene therapy [21]. Applications of polyomavirus-derived VLPs in diagnostics, vaccine development and gene delivery have been recently reviewed [22]. One human polyomavirus, the JC virus (JCPyV), can be found in the peripheral lymphocytes of healthy individuals [23] and has been shown to infect and replicate in B cells and to potentially become latent in the infected B cells [24]. The major JCPyV structural protein VP1 can self-assemble into a VLP structure when expressed in *E. coli* [25], yeast [26], or insect cells [27] and has a non-sequence-specific DNA-binding property [28]. It has recently been demonstrated that the JCPyV VLP was able to package plasmid DNA of larger size [21] and achieve higher gene transfer efficiency [29] when an *E. coli*–based *in vivo* packaging system was used. Therefore, it should be possible to use JCPyV VLPs to deliver genes of interest to tissue types that are the natural hosts of this virus for gene therapy purposes.

Recent research has revealed the presence of JCPyV DNA and protein in DLBCL tissues of the gastrointestinal tract [30], indicating that DLBCL cells are susceptible to JCPyV infection. Therefore, the JCPyV VLP may be able to deliver genes into human DLBCL cells for therapeutic purposes. In this study, we examined the ability of JCPyV VLPs to deliver either a reporter gene or a suicide gene to DLBCL cells and bring about the expression and functional effects of the gene in the transduced cells. We further assessed the ability of suicide gene–carrying JCPyV VLPs to target human DLBCL tumors in a xenograft animal model and inhibit the tumors’ growth, in order to gauge the potential of the JCPyV VLP to serve as a gene therapy vector for human DLBCL.

## Methods

### Cell lines

Human GCB-like DLBCL, Toledo (CRL-2631) and HT (CRL-2260), cell lines were purchased from the Bioresource Collection and Research Centre (Hsinchu, Taiwan). ABC-like DLBCL, SU-DHL-2 (CRL-2956), was purchased from ATCC (Manassas, VA). Cells were maintained in RPMI 1640 medium containing 2 mM L-glutamine, 1.5 g/L sodium bicarbonate, 4.5 g/L glucose, 10 mM HEPES, and 1 mM sodium pyruvate and supplemented with 10% fetal bovine serum (hereinafter referred to as complete culture medium).

### Preparation of green fluorescent protein–VLPs (gfp-VLPs) and thymidine kinase–VLPs (tk-VLPs)

JCPyV VLPs in which plasmids expressing green fluorescent protein (pEGFP-N3; BD Biosciences Clontech, CA) and thymidine kinase (pUMVC1-tk; Aldevron, ND) were packaged were prepared as described by Chen *et al.* [29]. In short, the above plasmids were propagated in and packaged into VLPs in *E. coli* that expressed JCPyV VP1, and the VLPs were purified from the *E. coli* lysates by 20% sucrose cushion and CsCl velocity gradient centrifugations. The fractions collected were dialyzed against Tris-buffered saline (10 mM Tris–HCl, pH 7.4, 150 mM NaCl) and analyzed for VLP content by the hemagglutination method, and the VLPs were concentrated by using Centricon filters (Millipore, Billerica, MA). VLPs packaged with pEGFP-N3 or pUMVC1-tk were named gfp-VLPs or tk-VLPs, respectively.

### Pseudoinfection of human DLBCL cells with gfp-VLPs

DLBCL cells were washed twice with phosphate-buffered saline (PBS), suspended in 50 μl PBS, and incubated with 10 μg of gfp-VLPs for 1 h at 4°C. Afterward, the cells were washed twice with cool PBS and cultured in complete culture medium at 37°C and 5% CO_2_ for 72 h. Green fluorescent protein expression was detected with a confocal microscope (LSM 510, Carl Zeiss, Thornwood, NY).

### Analysis of cytotoxicity of tk-VLPs in human DLBCL cells

The growth-inhibiting effect of tk-VLPs on Toledo cells was assessed with the MTT assay as follows. Toledo cells cultured in 96-well flat-bottom microtiter plates (BD Biosciences Clontech, San Diego, CA) at 1 × 10^4^ cells per well in complete culture medium were pseudoinfected with tk-VLPs at 1 μg per well. After gancyclovir (GCV) (Cymeven; Roche, Palo Alto, CA) was added to a final concentration of 10 μg/ml to a subset of the wells, leaving other wells without GCV as a control, the cells were incubated at 37°C with 5% CO_2_. At 2, 3, 4, and 5 days after pseudoinfection, the cells were collected by centrifugation, washed twice with PBS, suspended in complete culture medium containing 100 μl of 5 mg/ml 3-(4,5-dimethylthiazol-2-yl)-2,5-diphenyltetrazolium bromide (MTT, a tetrazole; Sigma, St. Louis, MO), and then cultured at 37°C with 5% CO_2_ for 1 h. Afterward, the cells were centrifuged to facilitate the removal of the medium and additives, 100 μl of dimethyl sulfoxide was added to the wells and allowed to incubate for 30 min, and the absorbance of each well was then measured in a spectroscope (Biotek Instruments, Winooski, VT) using a 595 nm filter.

### Analysis of human DLBCL tumor targeting by gfp-VLPs in a SCID mouse model

Four-week-old male SCID mice were purchased from the BioLASCO Taiwan Co., Ltd. (Taipei, Taiwan) and housed and maintained in controlled, specific pathogen–free airflow cabinets. The mice were given water and standard chow *ad libitum* and kept on a 12 h light/dark cycle. All animal procedures were performed according to approved protocols and in compliance with the recommendations for proper care and use of laboratory animals of the Institutional Animal Care and Use Committee of National Chung Cheng University.

Ten million Toledo cells were subcutaneously inoculated into each SCID mouse. After 1 month, the Toledo tumor–bearing mice were intravenously injected with gfp-VLPs at 105 μg per injection every three days, six times in total. The VLP inoculation dosage was determined by titration at the beginning of the study. After the entire course of VLP treatment was completed (18 days after the first VLP injection), the mice were anesthetized and their tumor nodules were removed and embedded in optimum cutting temperature compound (Sakura Finetek USA, Inc., Torrance, CA). Frozen section was performed to give slices of 6 um thickness. Green fluorescent protein expression was detected with a ZEISS AXioskop2 upright fluorescence microscope (Carl Zeiss, Thornwood, NY).

### Analysis of inhibition of human DLBCL tumor growth by tk-VLPs in a SCID mouse model

To induce the formation of human DLBCL tumor nodules in mice, Toledo cells were harvested from subconfluent cultures, washed once with a serum-free medium, resuspended in PBS, and injected subcutaneously into the right flank of SCID mice at 1 × 10^7^ cells per mouse. One month later, the Toledo tumor–bearing mice were randomized into four groups of four mice each and subjected to different treatment combinations: tk-VLPs with or without GCV; control (non-transgene-carrying) VLPs with or without GCV. tk-VLPs or control VLPs were administered intravenously at 105 μg per injection every three days, three times in total. GCV was administered by intraperitoneal injection (300 mg kg^−1^) every three days after the first tk-VLP or control VLP injection. The mice were euthanized when the tumors reached a volume of approximately 10,000 mm^3^, and the tumors were removed and weighed.

### Statistical analysis

Data were expressed as mean ± standard deviation. Data analysis was performed using Student’s *t-*test and by one- or two-way ANOVA, with a *P*-value <0.05 being considered to represent a significant difference.

## Results

### Confirmation of reporter gene transfer by JCPyV VLPs into DLBCL cells *in vitro* and in a heterotopic xenograft mouse model

**Figure 1:**
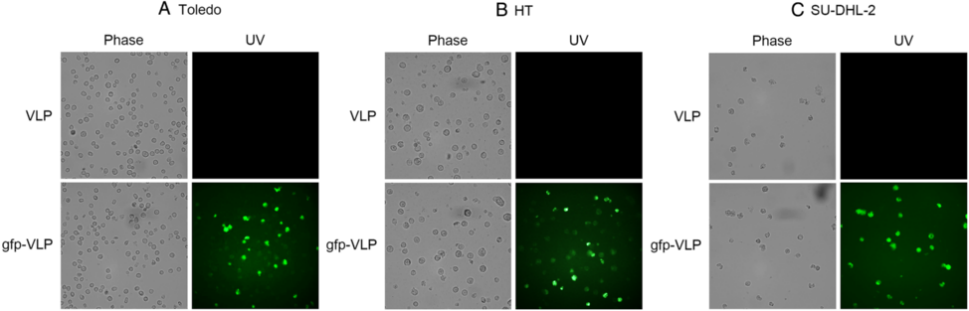
**Transduction of the green fluorescent protein gene into human DLBCL cells by JCPyV VLPs** ***in vitro*** **.** GCB-like, Toledo **(A)** and HT **(B)**, and ABC-like, SU-DHL-2 **(C)**, DLBCL cells were infected with control VLPs or with gfp-VLPs. The expression of green fluorescent protein in the infected cells was visualized with a fluorescence microscope.

**Figure 2:**
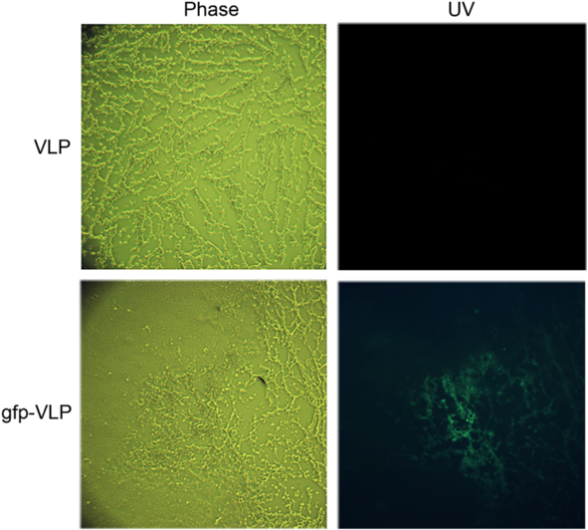
**Transduction of the green fluorescent protein gene into human DLBCL tumor nodules by JCPyV VLPs in a xenograft mouse model.** Human DLBCL–xenografted mice were administered control VLPs or gfp-VLPs intravenously. The DLBCL tumor nodules were frozen-sectioned, and the expression of green fluorescent protein was visualized with a fluorescence microscope.

In order to evaluate the suitability of JCPyV VLPs as gene transfer vectors for DLBCL therapy, we first needed to determine if the VLPs can enter DLBCL cells just as native JCPyV virions can. Thus, we tested JCPyV VLPs carrying the green fluorescent protein gene, or gfp-VLPs, for gene transduction activity using Toledo and HT cells (GCB-like) and SU-DHL-2 (ABC-like) as cell models. DLBCL cells in culture were treated with gfp-VLPs, and reporter gene expression was assessed 72 h later by examination under a fluorescence microscope. As shown in Figure 1, JCPyV VLPs not only entered the target cells but also transferred the green fluorescence gene to the cells and led to its expression, indicating the feasibility of using these VLPs as vectors for exogenous gene delivery to both GCB-like and ABC-like DLBCL cells for expression. As any future therapeutic application of JCPyV VLPs requires their effectiveness to be demonstrated in an animal model, we next tested whether the VLPs can reach DLBCL tumors in living animals via blood circulation, by using severe combined immunodeficiency (SCID) mice as an animal model. SCID mice were subcutaneously injected in the flank with Toledo cells, and after solid tumors had formed, the mice were injected through the tail vein with gfp-VLPs every three days for three cycles. Afterwards, the tumor nodules were removed, embedded in optimum cutting temperature compound, frozen-sectioned, and examined under a fluorescence microscope. As shown in Figure 2, JCPyV VLPs that were introduced into the blood circulation not only gained entry into the Toledo tumor nodules but also transferred the green fluorescence gene to the tumor cells, resulting in the proper expression of the gene therein. These results show that JCPyV VLPs are able to effectively deliver the exogenous genes they carry to human DLBCL cells for expression.

### Cytotoxic activity of combined tk-VLPs and ganciclovir (GCV) in DLBCL cells *in vitro*

**Figure 3:**
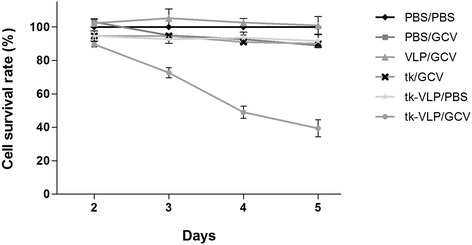
**Cytotoxic effect of tk-VLPs on human DLBCL cells.** The viability of DLBCL cells at different days after various treatments was assessed by the MTT assay. The treatment combinations included PBS followed by PBS (PBS/PBS), PBS followed by GCV (PBS/GCV), control VLPs followed by GCV (VLP/GCV), the pUMVC1‐tk plasmid followed by GCV (tk/GCV), tk-VLPs followed by PBS (tk‐VLP/PBS) and tk-VLPs followed by GCV (tk-VLP/GCV).

The DLBCL treatment strategy currently in clinical use (R-CHOP) promises greater therapeutic success by combining several chemotherapeutic agents with a monoclonal antibody, but the efficacy of this therapy has been limited by chemoresistance arising from the genetic heterogeneity of the tumor cell populations. For this reason, we chose to exploit the higher proliferative activity of all tumor cells relative to normal cells by employing the suicide gene therapy approach with the JCPyV VLP vectors. Our chosen therapeutic gene, HSV-1 thymidine kinase, is a nucleoside kinase that, by adding a phosphate group to the prodrug GCV and consequently disrupting chain elongation during DNA replication, serves as a suicide gene for actively replicating tumor cells, with relatively little effect on normal cells. We purified JCPyV VLPs packaged with the thymidine kinase gene, or tk-VLPs, and assayed them in combination with GCV for cytotoxicity in human DLBCL cells *in vitro*. As shown in Figure 3, the growth of DLBCL cells was increasingly inhibited with increasing days of treatment with combined tk-VLPs and GCV, reaching an inhibition rate of 60% after 5 days of treatment relative to the control treatment with PBS. No growth inhibition was observed with tk-VLP or GCV treatment alone. These results show that JCPyV VLPs are able to deliver the thymidine kinase gene to human DLBCL cells for expression and produce a strong cytotoxic effect in combination with GCV.

### Inhibition of human DLBCL growth by tk-VLPs in a xenograft mouse model

**Figure 4:**
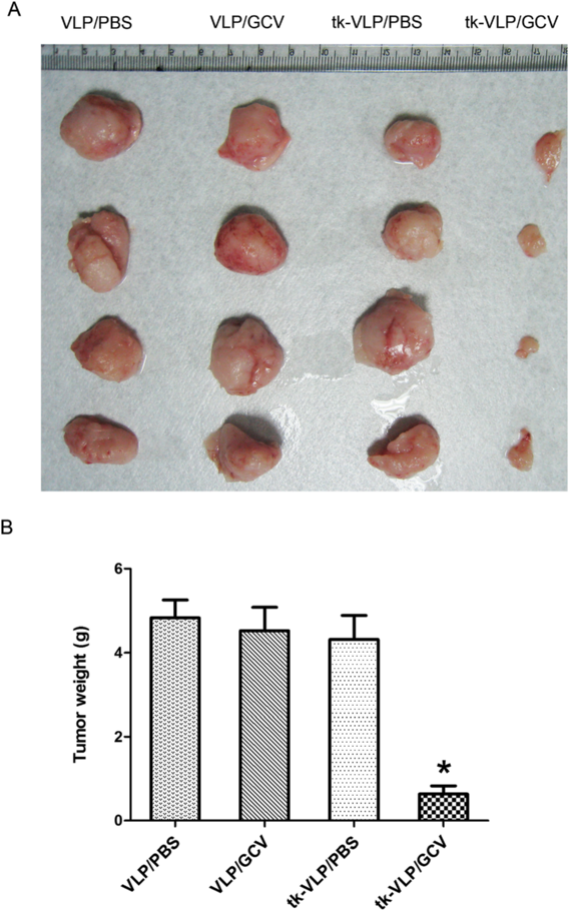
**Inhibition of human DLBCL tumor nodule growth by tk-VLPs in a xenograft mouse model.** The human DLBCL–xenografted mice were intravenously administered control VLPs or tk-VLPs in the presence or absence of GCV. **(A)** Gross pictures of tumor nodules from each treatment group. **(B)** Quantification of tumor weights for the different treatment groups. *, P < 0.05.

After confirming the growth-inhibiting effect of our suicide gene approach on human DLBCL cells *in vitro,* we further assessed whether the tk-VLP treatment is similarly effective *in vivo* and thus potentially therapeutically useful*.* As before, we used SCID mice as an animal model by subcutaneously implanting Toledo human DLBCL cells into the mice, and began the testing of tk-VLPs one month later when tumor nodules had formed. tk-VLPs were injected into the mice intravenously to allow the VLPs to reach the DLBCL tumors via blood circulation and transduce the target cells with the thymidine kinase gene; GCV was injected into the mice the next day to activate the cytotoxic activity of the suicide gene, completing the first cycle of treatment. This treatment cycle was repeated every three days for a total of three cycles, after which the subcutaneous nodules were removed and weighed to measure the therapeutic effect of the tk-VLP/GCV combination. As revealed by our results, the size of the tumor nodules at the end of the treatment course averaged more than 4 g for all control treatment groups, but was on average less than 1 g for the tk-VLP/GCV combination group (Figure 4A,B), amounting to a rate of tumor growth inhibition of at least 80% for the combination treatment. Collectively, our results show that our JCPyV VLP vector and suicide gene therapy strategy can indeed inhibit the growth of DLBCL tumors in a highly effective manner both *in vitro* and *in vivo*, indicating the potential of our novel system to become a useful treatment strategy for DLBCL.

## Discussion

In this study, we demonstrate both *in vitro* and *in vivo* that JCPyV VLPs can enter human DLBCL cells and express the exogenous genes they carry with high efficiency, as evidenced by the expression of the green fluorescent reporter, and can deliver the thymidine kinase suicide gene into DLBCL cells to induce cell death in the presence of GCV. In our animal model, tk-VLPs injected through the tail vein were able to reach xenografted DLBCL tumor nodules via blood circulation and induce cytotoxicity in the nodules in the presence of GCV, resulting in a drastic 80% reduction in tumor growth. These results strongly support the promise of tk-VLPs as a gene therapy strategy for DLBCL.

DLBCL is the most common, aggressive subtype of B-NHL [31] and is conventionally treated with CHOP chemotherapy. While the majority of patients initially respond well to CHOP, approximately 50% of the patients eventually relapse and will die without timely transplantation [32]. DLBCL patients differ widely in sensitivity to chemotherapy, which can be attributed to the genetic heterogeneity of DLBCLs [33], and chemoresistance in lymphomas can involve not only the overexpression of the MDR multidrug pump but also certain molecular pathways [34,35]. One known pathway is the PI3K/AKT signaling pathway, which plays an important role in the proliferation and chemoresistance of non-Hodgkin lymphomas [36,37]. Deregulation of this pathway is often observed in DLBCLs and has been associated with poor prognosis [38-40]. Because of the important role mTOR plays in the AKT pathway, it has become a therapeutic target in DLBCL treatment [41]. In a phase II clinical trial, the mTOR inhibitor everolimus was found to produce an overall response rate of 30% in relapsed patients [42]. Overexpression of antiapoptotic proteins of the BCL-2 family has also been linked to chemotherapy resistance in B-cell lymphoma [43,44]. BCL-2 overexpression was detected in 20% of DLBCL patients [45,46] and has been associated with a higher relapse rate and shorter disease-free survival [47,48]. In a phase II study, a BCL-2–specific antisense oligonucleotide in combination with rituximab achieved an overall response rate of 42% in B-NHL patients [49]. In addition, activation of Src family kinases (SFK) is also known to induce B cell activation and survival [50,51]. The active form of SFK was found in over a third of DLBCL patients, suggesting the possibility of treating relapsed B-NHL patients with the SFK inhibitor dasatinib [52]. The above-mentioned molecular pathways alone point to the highly heterogeneous nature of DLBCLs, and yet other pathways have been shown to lead to drug resistance in B-NHL [53]. Thus, agents against new molecular targets and multitargeted combinatorial therapies may offer new hope for drug-resistant relapsed patients. It has been reported that JCPyV VLPs are able to package and deliver small molecules to the target cells for therapeutic purposes [54-56]. Therefore, it is also possible to package and deliver an antisense oligonucleotide or siRNA against BCL-2 or small molecule drugs, such as everolimus and dasatinib, to diminish drug-resistant relapse of DLBCL.

Suicide gene therapy generally consists of two steps: an exogenous enzyme-expressing gene is first transferred to tumor cells via a vector; an inactive prodrug is then administered that is converted by the exogenous enzyme into a lethal drug, which kills the tumor cells [57]. A number of prodrug activation systems are being studied, such as the GCV-converting HSV thymidine kinase (HSV-TK) [58]; cytosine deaminase, which converts the nontoxic 5-fluorocytosine into 5-fluorouracil [59]; *E. coli* purine nucleoside phosphorylase with its prodrug substrate 6-methylpurine-2′-deoxynucleoside [60]; nitroreductase/5-(aziridin-1-yl)-2,4-dinitrobenzamide (CB1954) [61]; and linamarase/linamarin [62]. Among the most promising of suicide gene therapy systems is HSV-TK/GCV [63], which is cell cycle–dependent and affects only dividing cells, a particularly advantageous feature for cancer therapy applications. When expressed in cells, HSV-TK metabolizes GCV to GCV monophosphate, which is further phosphorylated by endogenous cellular kinases to form the toxic metabolite GCV triphosphate [64]. GCV triphosphate inhibits DNA polymerase and causes DNA chain termination, thereby blocking DNA replication and inducing tumor cell death. It has been shown that incorporation of GCV triphosphate into DNA leads to cell cycle arrest in the S and G2 phases and consequently apoptosis [65]. The antitumor activity of the HSV-TK/GCV gene therapy system has been demonstrated in many animal models of cancer, including leukemia [66], glioma [67], bladder cancer [68], and colon adenocarcinoma [29]. These promising findings have resulted in pre-clinical studies of this therapy system for various types of cancer [69-72] and suggests its potential applicability in the treatment of B-NHL.

Recent research on JCPyV VLPs has shown them to be promising gene therapy vectors [73]. JCPyV VLP–mediated gene transfer has been used to induce apoptosis in polyomavirus-transformed cells [56], and to inhibit native virus replication in JCPyV-susceptible cells [54]. Besides their broad applicability in gene therapy, JCPyV VLP vectors also have the advantage of being relatively easy to prepare and safe. JCPyV VLPs can be produced in an *E. coli* expression system on a large scale at low cost [25], and the *in vivo* DNA packaging method developed for them [29], which replaces *in vitro* osmotic shock [74], not only increases their packaging efficiency but also eliminates the risk of viral virulence from packaging the viral genome, as well raising the size limit of the packaged DNA fragment to 9.4 kbp [21]. All the above evidence suggests that JCPyV VLPs make excellent gene delivery vectors. Therefore, in this study, we used JCPyV VLPs as the vectors for carrying out the HSV-TK/GCV system, exploiting the intrinsic ability of the VLPs to seek out naturally susceptible cells *in vivo*. We found that tk-VLPs introduced into mice by tail vein injection were indeed able to specifically target subcutaneous human DLBCL nodules and cause them to shrink (Figure 4), but mouse cells are not susceptible to JCPyV VLPs infection. These results indicate that the JCPyV VLPs were able to protect their packaged HSV-TK DNA during transit in the systemic circulation and deliver the DNA to the target DLBCL cells for expression. JCPyV VP1 contains three external loops in its surface structure [73,75]. The surface loops may be responsible for immune recognition. Therefore, modification of the surface domains of JCPyV VLPs may avoid immune recognition in the future.

## Conclusions

In summary, we have demonstrated here that the JCPyV VLP can be used as a gene delivery vector for gene therapy for human DLBCL both *in vitro* and *in vivo*. Although many aspects remain to be improved before practical application, tk-VLPs have the potential to become a therapeutic choice for human B-cell lymphomas in the future.

## Abbreviations

DLBCL: Diffuse large B-cell lymphoma
GCB-like DLBCL: Germinal center B-cell-like DLBCL
ABC-like DLBCL: Activated B-cell-like DLBCL
JCPyV: JC Polyomavirus
VLPs: Virus-like particles
TK: Thymidine kinase
GCV: Ganciclovir
B-NHL: B-cell non-Hodgkin lymphoma
MDR: Multidrug resistance
HSV: Herpes simplex virus
